# Capturing Relevant Patient Data in Clinical Encounters Through Integration of an Electronic Patient-Reported Outcome System Into Routine Primary Care in a Boston Community Health Center: Development and Implementation Study

**DOI:** 10.2196/16778

**Published:** 2020-08-19

**Authors:** Stephanie Loo, Chris Grasso, Jessica Glushkina, Justin McReynolds, William Lober, Heidi Crane, Kenneth H Mayer

**Affiliations:** 1 The Fenway Institute Boston, MA United States; 2 Clinical Informatics Research Group Biobehavioral Nursing and Health Informatics University of Washington Seattle, WA United States; 3 Division of Allergy and Infectious Diseases Department of Medicine University of Washington Seattle, WA United States; 4 Harvard Medical School Boston, MA United States; 5 HIV Prevention Research Beth Israel Deaconess Hospital Boston, MA United States

**Keywords:** information technology in health, primary care, technology adoption, technology diffusion

## Abstract

**Background:**

Electronic patient-reported outcome (ePRO) systems can improve health outcomes by detecting health issues or risk behaviors that may be missed when relying on provider elicitation.

**Objective:**

This study aimed to implement an ePRO system that administers key health questionnaires in an urban community health center in Boston, Massachusetts.

**Methods:**

An ePRO system that administers key health questionnaires was implemented in an urban community health center in Boston, Massachusetts. The system was integrated with the electronic health record so that medical providers could review and adjudicate patient responses in real-time during the course of the patient visit. This implementation project was accomplished through careful examination of clinical workflows and a graduated rollout process that was mindful of patient and clinical staff time and burden. Patients responded to questionnaires using a tablet at the beginning of their visit.

**Results:**

Our program demonstrates that implementation of an ePRO system in a primary care setting is feasible, allowing for facilitation of patient-provider communication and care. Other community health centers can learn from our model in terms of applying technological innovation to streamline clinical processes and improve patient care.

**Conclusions:**

Our program demonstrates that implementation of an ePRO system in a primary care setting is feasible, allowing for facilitation of patient-provider communication and care. Other community health centers can learn from our model for application of technological innovation to streamline clinical processes and improve patient care.

## Introduction

Electronic patient-reported outcome (ePRO) systems can improve health outcomes by detecting health issues or risk behaviors that may be missed when relying on provider elicitation [1]. The use of computerized assessments has the potential to further improve patient-provider communication and overall satisfaction though systematic data collection [2]. The ePRO system was developed for research in 2007, through the Center for AIDS Research Network of Integrated Clinical Systems’ collection of HIV-specific PROs [3,4]. Fenway Health (hereafter, referred to as “Fenway”) is a national leader in HIV care, research, and culturally responsive care to lesbian, gay, bisexual, transgender, and queer (LGBTQ) patients [5]. Fenway serves a diverse group of around 30,000 patients, more than 17,000 of whom identify as LGBTQ, over 2000 of whom are persons living with HIV, and 30% comprise racial and ethnic minorities. The ePRO integration project was implemented across all primary care clinic sites of Fenway, an urban federally qualified community health center. The pilot project began in 2013, with full implementation and rollout occurring across three primary care clinic sites at Fenway from 2014 to 2015.

An average primary care patient is due for 25 different services at the time of the visit [6], resulting in increased paperwork and data entry burden for staff [7]. Innovations such as the ePRO system at Fenway increase clinical efficiency and reduce both patient and provider burden during clinic visits, allowing providers to focus on the most salient aspects of the patient’s reason of visit. An ePRO system that administers key health questionnaires was implemented at Fenway and integrated with the electronic health record (EHR), allowing medical providers to review and adjudicate patient responses in real-time during medical visits. The goal of this ePRO project was to implement and evaluate the ePRO interface for all patients accessing primary care at a Boston community health center.

## Methods

### Development of Technology and Clinical Processes

We developed a graphical user interface and underlying algorithm to populate a set of assessments based on how recently the previous assessment was completed, if ever; clinical priority (eg, repeat depression screen for patient with a depression diagnosis); active diagnoses; and estimated time to complete the assessment*.*

**Figure 1:**
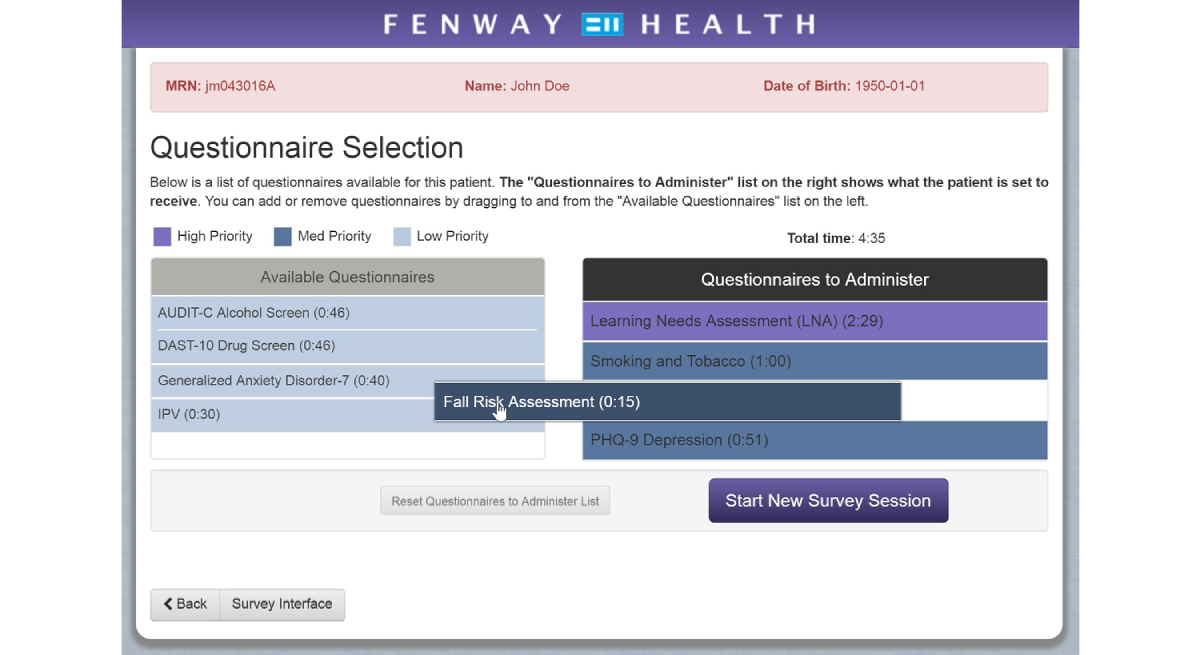
Fenway’s electronic patient-reported outcome (ePRO) survey interface.

Patients received a tablet device containing validated surveys focusing on key health domains such as Patient Health Questionnaire-9 (PHQ-9) depression scale and the Alcohol Use Disorders Identification Test (AUDIT) during their medical visit. Fenway customized the ePRO platform to alert providers if patients reported suicidal ideation (a positive response to Question 9 of the PHQ-9 depression screen [8]). Fenway began with 4 assessments and eventually expanded to 10, addressing behavioral health, substance use, and fall risk. As more assessments were added to the ePRO interface, the underlying algorithm for determining priority and frequency of each assessment was refined. Features of the selection menu included automated color coding and ranking of prioritized assessments, limiting total assessments to a 5-minute timeframe. In addition, clinical staff could select any or all available assessments for administration, depending on clinical need and available time for the patient to take the assessments. Estimated time to complete designations was generated for each survey based upon the total number of potential questions within each survey and running mock-survey sessions by the program team. The design of the assessments included multiple-choice, check box, fill-in-the-blank, and drop-down questions based on prior responses (Figure 1). The available surveys were, namely, learning needs assessment, PHQ-9/PHQ-9 modified for Adolescents (PHQ-A), smoking and tobacco, fall risk assessment, intimate partner violence, AUDIT-C alcohol screen, Drug Abuse Screening Test-10 (DAST-10), Generalized Anxiety Disorder-7 (GAD-7), Edinburgh postpartum screen, patient portal sign-up.

The platform was configured to determine which surveys were due for each patient, including logic to create skip patterns depending on a patient’s response. Using Health Level 7 standards, the platform interfaced with Fenway’s EHR (Centricity Practice Solutions). As such, patient responses automatically uploaded as structured data into their medical record for provider review, decreasing data entry errors and improving workflow efficiency and accuracy. The data were also sent to the EHR as a summary report in PDF format with score interpretations, making review of the data simple. Time from patient completion of assessments to results appearing in their medical record was approximately 2 minutes.

**Figure 2:**
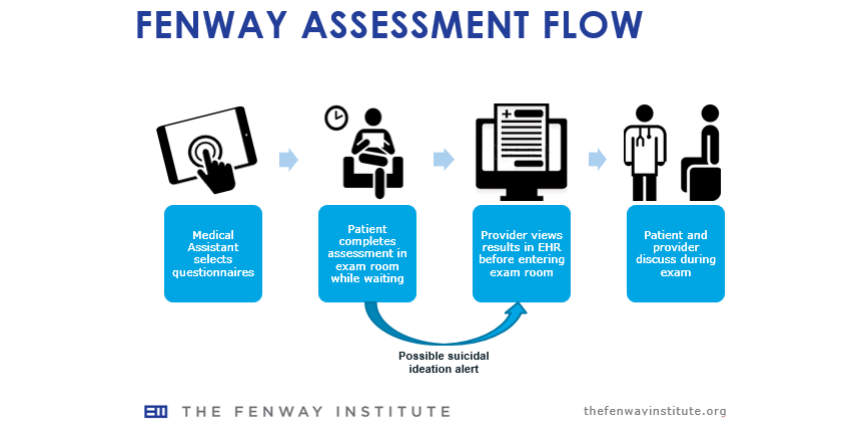
Visualization of electronic patient-reported outcome (ePRO) workflow in Fenway’s medical department.

Patients received a tablet device containing validated surveys at the beginning of their medical visit prior to their medical provider entering the room. Patients filled out the assessments in the exam room after receiving the tablet from a medical assistant. The exam room was chosen over the waiting room area due to patients’ shorter wait times as well as to allow for privacy should a patient have any questions for the medical assistant or provider. The platform was configured to determine which assessments were due for each patient (Figure 2), allowing for a dynamic system that would customize order and prioritize assessments to individual patient diagnoses and needs.

The suggested time limit of 5 minutes was generated based on the discussion with clinical leadership during the preproject implementation. However, the interface allowed medical staff to select and choose preferred assessments tailored to individual patient need and available waiting time. For example, if a patient arrived and was roomed early for their appointment, the medical assistant could select more assessments beyond the 5-minute time limit for the patient to complete. If the provider could see the patient earlier, the medical assistant could select one or two prioritized surveys that would take less time for the patient to complete and not impede upon their clinical visit time.

### Evaluation of ePRO Implementation

We conducted two brief mixed methods evaluations during the implementation to ascertain provider and patient feedback on the ePRO system. A paper-based survey was sent out to providers and medical assistants in early 2016 to ask how the program was working for them. A focus group with Fenway primary care patients was held in July 2016 to generate patient feedback on the ePRO program. As these activities were part of clinical quality improvement activities, Institutional Review Board approval was not necessary.

### Costs

This work was made possible through a one-time 2-year grant provided by Neighborhood Health Plan and the Partnership for Community Health’s *Excellence and Innovation* program. This funding primarily covered costs of a full-time program manager to design; implement; and oversee the ePRO program as well as those of equipment needs, including tablets, storage lockers, protective cases, and sanitation wipes for cleaning.

## Results

### Development of Technology and Clinical Processes

Piloting began in 2013, with implementation and rollout occurring across three primary care clinical sites from 2014 to 2015. The ePRO system was initially piloted with 5 medical assistants serving 3 primary care providers, and eventually expanded to 18 medical assistants and 25 primary care providers across three clinical sites in the first year of expansion within the Fenway medical department. Completion rates of ePRO sessions grew significantly over time, from 2428 completed sessions in 2014 to 19,650 completed sessions in 2018. We noted an increase in the total number of ePRO sessions as well as the number of ePROs taken by the same patient, demonstrating increased and repetitive use of the interface as patients return for follow-up care. From 2016 to 2017, the percentage of patients who took and completed at least one assessment using the ePRO system increased from 66% to 74%. In 2018, via ePRO, 41% of patients reported mild-to-severe depression, 35% reported mild-to-severe anxiety, 35% reported problem alcohol use, 4.4% reported a positive DAST score, and 10% reported current or some-day tobacco use (Table 1). In 2018, there were 300 suicide ideations alerts from the ePRO platform, wherein a suicide ideation response of “Nearly Every Day” comprised 1.5% of total ePRO sessions in the same year.

**Table 1:** Electronic patient-reported outcome (ePRO) prevalence data for 2016-2018.

Condition	Instrument	Prevalence, n/N (%)		
		2016	2017	2018
Mild-to-severe depression	PHQ-9^a^	6644/13899 (47.80)	8469/16237 (52.16)	9022/15857 (56.90)
Mild-to-severe anxiety	GAD-7^b^	2748/6813 (40.33)	4870/10542 (46.20)	7664/14456 (53.02)
Problem alcohol use (high risk - likely addiction)	AUDIT^c^	115/5365 (2.14)	195/8785 (2.22)	340/14345 (2.37)
Moderate-to-severe drug use	DAST-10^d^	178/5438 (3.27)	174/8988 (1.94)	641/14470 (4.43)
Current or some-day smoker	Smoking & Tobacco	994/8736 (11.38)	1333/14164 (9.41)	1527/14992 (10.19)

^a^PHQ-9: Patient Health Questionnaire-9.

^b^GAD-7: Generalized Anxiety Disorder-7.

^c^AUDIT: Alcohol Use Disorders Identification Test.

^d^DAST-10: Drug Abuse Screening Test-10.

### Evaluation of ePRO Implementation

Primary care providers, medical assistants, and patients were generally satisfied with the ePRO program. A survey of primary care providers was conducted in early 2016, wherein 75% (27/36) providers responded. A total of 70.4% (19/20) of medical providers agreed/strongly agreed that use of the ePRO system improved access to real-time data in patient medical charts. Similarly, 66.7% (18/27) indicated that using the ePRO system reduced paperwork burden. In a patient focus group (n=8) conducted in July 2016, patients appreciated how the technology facilitated quick responses from providers and saved time during their medical appointments. Patients reported that the program allowed them to answer challenging questions in a nonstigmatized manner, which they found valuable, particularly when meeting a provider with whom they had no prior relationship. Patients noted that the ePRO system made them feel like direct participants in their medical care.

### Costs

The grant used to fund staff and equipment purchases assisted with program start-up costs. As the ePRO program expanded and became highly accepted and endorsed by medical staff, associated costs with maintaining the equipment used were absorbed into health center operation budgets. These associated costs were minimal, as they primarily covered purchases of sanitation wipes for tablet cleaning between patients. The program manager who initially oversaw the project was transferred. The overall supervision of the ePRO program remained in place for troubleshooting any issues but the project manager moved on to other projects due to the reduction of needed full-time equivalent (FTE) units following successful implementation of the system.

## Discussion

Fenway’s ePRO program is highly accepted by both clinical staff and patients. Fenway recently developed other ePRO programs for the behavioral health and patient registration departments following the success of the program in primary care. The patient registration ePRO department uses interactive registration forms to collect PRO data from patients in the waiting room. While this program initially received a grant aimed at implementation of PROs that covered high early start-up costs, Fenway found that these costs were absorbed over time, as equipment needs did not change and initial staff management of ePRO became embedded in clinical workflow. Further, successful implementation of the program resulted in multiple benefits for the health center in saved paperwork and data entry time as well as helping the health center meet PRO-related quality measures and fee-for-service benchmarks. Achieving sustainability for this project required buy-in from health center and departmental leadership, in addition to establishing an ePRO process that did not disrupt clinical workflows.

The primary challenge in implementing the ePRO program centered on overcoming hesitations of primary care providers and medical assistants at implementing a new program within the existing clinical workflows. This reluctance dissipated as clinic teams observed how colleagues participating in the pilot program saved time, as the program eliminated the need to print and hand out health questionnaires before patient visits and to input patient responses in patient charts afterwards. The program manager also met one-on-one with primary care providers and medical assistants to train them on the new system and any changes to clinic forms. This hands-on approach to building rapport combined with frequent solicitation of clinical staff feedback assisted with gaining staff buy-in.

The routine integration of PROs into clinical care can have many potential advantages, including improving patient-provider communication, improving care, and facilitating research. Patient care can be improved by enabling clinicians to address functional problems, mental health problems, or symptomatic conditions that might otherwise be missed. The ePRO system at Fenway proves to be an acceptable, sustainable method of quickly assessing patients, although further work needs to understand the true effects of ePRO on clinical care and improvement in a primary care setting.

## Abbreviations

AUDIT: Alcohol Use Disorders Identification Test
DAST: Drug Abuse Screening Test
EHR: electronic health record
ePRO: electronic patient-reported outcome
FTE: full-time equivalent
GAD-7: Generalized Anxiety Disorder-7
LGBTQ: lesbian, gay, bisexual, transgender, and queer
PHQ-9: Patient Health Questionnaire-9
PHQ-A: PHQ-9 modified for Adolescents
